# Short-Term Lifestyle Strategies for Sustaining Cognitive Status

**DOI:** 10.1155/2016/7405748

**Published:** 2016-11-07

**Authors:** Elizabeth P. Howard, John N. Morris, Knight Steel, Kelley A. Strout, Brant E. Fries, Alice Moore, Vjenka Garms-Homolová

**Affiliations:** ^1^Bouvé College of Health Sciences, School of Nursing, Northeastern University, 360 Huntington Avenue, Boston, MA 02115, USA; ^2^Hebrew SeniorLife, Institute for Aging Research, 1200 Centre Street, Boston, MA 02131, USA; ^3^Hackensack University Medical Center, 30 Prospect Avenue, Hackensack, NJ 07601, USA; ^4^School of Nursing, University of Maine, 5724 Dunn Hall, Orono, ME 04469, USA; ^5^Institute of Gerontology and Geriatric Research, Education and Clinical Center, Ann Arbor VA Healthcare Center, University of Michigan, 300 NIB 933 NW, Ann Arbor, MI 48109, USA; ^6^Nurse Practitioner Professional Resources, 4361 Route 42, Turnersville, NJ 08012, USA; ^7^Hochschule für Technik und Wirtschaft, Hönower Straße, 10318 Berlin, Germany

## Abstract

Cognitive decline impacts older adults, particularly their independence. The goal of this project was to increase understanding of how short-term, everyday lifestyle options, including physical activity, help an older adult sustain cognitive independence. Using a secondary analysis of lifestyle choices, we drew on a dataset of 4,620 community-dwelling elders in the US, assessed at baseline and one year later using 2 valid and reliable tools, the interRAI Community Health Assessment and the interRAI Wellness tool. Decline or no decline on the Cognitive Performance Scale was the dependent variable. We examined sustaining one's status on this measure over a one-year period in relation to key dimensions of wellness through intellectual, physical, emotional, social, and spiritual variables. Engaging in physical activity, formal exercise, and specific recreational activities had a favorable effect on short-term cognitive decline. Involvement with computers, crossword puzzles, handicrafts, and formal education courses also were protective factors. The physical and intellectual domains of wellness are prominent aspects in protection from cognitive decline. Inherent in these two domains are mutable factors suitable for targeted efforts to promote older adult health and well-being.

## 1. Introduction

Cognitive decline is the most feared consequence of aging [1]. The loss of the ability to think clearly and process information correctly may change how older adults interface with almost all aspects of their environment, thereby impacting their independence and self-perception. As the older adult population increases, more attention is directed toward holistic health and wellness choices that might sustain a person's cognitive status and support successful aging [2–6]. “Physical fitness” and “brain fitness” solutions have emerged as potentially valuable strategies for maintaining and improving cognitive functioning among older adults [7].

The goal of this project is to better understand how the short-term, everyday lifestyle options available to an older adult help to sustain cognitive independence as assessed by the widely used Cognitive Performance Scale (CPS) [8]. This scale is grounded on the person's ability to plan everyday tasks and to engage in meaningful communication with others. It has been shown to have a high correlation with the Mini Mental State Examination [8] and has been widely used [9–11]. Furthermore, the dimensions assessed by the CPS are quite relevant to older adults' continued control over everyday activities.

## 2. Factors Influencing Cognitive Decline

We examined cognitive decline from the perspective of independent variables arising from the dimensions of wellness model [12]. These include measures relative to physical, intellectual, emotional, social, occupational/leisure, and spiritual wellness. For several “dimensions,” the literature already supports an association between cognitive health protection and early lifestyle choices made by the person [13]. These choices are in addition to the person's avoidance of specific conditions. Much of this research, however, is based on earlier life choices. Perhaps equally important, there are indications that even an aged person can initiate lifestyle changes that may help to sustain their current level of cognitive performance [14–16].

The amount of time and intensity of engagement in physical exercise/activity throughout the lifespan are related to cognitive health [14, 17–19]. In one prospective study, persons who had exercised three or more times per week earlier in life were found to be less likely to develop dementia as they aged [20]. Results from Dik and colleagues [18] demonstrated a similar lifetime protective role. Of particular interest, Angevaren and colleagues [17] found that those who increased or maintained the intensity of physical activity earlier in life were more likely to demonstrate a stronger cognitive performance when older as compared to those who engaged in a diminishing intensity of physical activity. The same relationship has been found among older individuals participating in the Rush Memory and Aging Project, a prospective observational cohort study. A higher level of daily physical activity among older persons was associated with a reduced risk of Alzheimer's disease [13]. Similarly, beneficial effects on memory function were found among older people after a six-month intervention of medium and even low-intensity physical activity versus control [21]. Aerobic exercise when combined with strength and flexibility training demonstrated benefits in cognitive function that was enhanced if the exercise involved other tasks such as coordination and scheduling [22].

Intellectual wellness demonstrated through an earlier life participation in cognitively stimulating activities or purposeful cognitive training appears to have a favorable effect on cognitive health [23, 24]. In a cross-sectional analysis of a sample of 145 persons between the ages of 70 and 91, Gilhooly et al. [23] found that stimulating activities such as computer games, crossword puzzles, and reading reduced the risk of cognitive health decline among those with limited formal education. Lachman et al. [24] reported similar results. Park and colleagues found engaging in productive activities and learning new, challenging skills improved episodic memory [25].

Socializing via activities based on the exchange of existing knowledge, for example, cooking, playing games, and watching movies, does not improve cognitive performance [25]. Emotional wellness and social engagement patterns in younger years have not been examined sufficiently to support their value as a predictor of cognitive health as compared to intellectual and physical wellness variables. Forstmeier and Maercker found that adults' lifetime motivational ability, choosing between alternative goals, and working toward achieving a chosen goal are associated with better cognitive health in old age [26]. By comparison, Wilson and colleagues (2007) carried out a twelve-year longitudinal study of individuals with high neuroticism scores at baseline and reported that 42% of these persons were more likely to develop cognitive impairment when compared to individuals with low neuroticism scores. Additionally, the risk of mild cognitive impairment increased by 6% for each depressive symptom [27].

Köhler et al. also found that depressive symptoms can increase the risk of cognitive decline [28]. In a cohort of 479 adults over age 60, those who demonstrated a high number of depressive symptoms at baseline were at significantly greater risk of developing cognitive impairment at a six-year follow-up as compared to those with no or fewer depressive symptoms. In another longitudinal analysis, Wang and colleagues found that persons with low neuroticism and high extraversion at baseline demonstrated the lowest risk for developing dementia at six-year follow-up [29].

The dimensions of physical wellness and social engagement and their inherent complexity lead one to consider the quantity and quality of sleep, cognitive stimulation, and brain exercises in relation to overall wellness. An association between sleep and cognition appears to exist. In one study examining change in cognition over six months, poor sleep quality was associated with cognitive decline [30]. Kociuba et al. (2010) reported similar findings [31]. In a population based cohort study of 4,010 adults, using data collected at baseline and 8.5 years later, Loerbroks et al. found that increases in nocturnal sleep duration were associated with cognitive impairment [32]. Similar results regarding the relationship between sleep duration and cognition were reported by Anguera and colleagues [33]. Other work points to quality of sleep. In the Bronx Aging Study, frequent interruptions of sleep and low sleep efficacy influenced cognitive performance [34] and nonrestorative sleep and excessive daytime sleepiness were identified as predictors of dementia [35].

Spiritual wellness is best defined as having purpose in life and a value system [12]. At a seven-year follow-up, older adults with high purpose in life were 2.4 times more likely to remain free of Alzheimer's disease and mild cognitive impairment as compared to adults with a low purpose in life [36] but findings in this area are not well substantiated.

The primary purpose of the research reported in this paper is to further understand how everyday intellectual, physical, and spiritual lifestyle choices by the older adult as well as the absence of life complications (e.g., depression) influence a short-term, one-year difference in sustaining cognitive ability as measured by the cognitive performance scale (CPS).

## 3. Material and Methods

The COLLAGE consortium in the US provided the settings for this project. As a nonprofit, national group of senior housing environments, it offers a comprehensive assessment system drawing on tools from the interRAI suite [9, 37, 38].

Conducting a secondary analysis of lifestyle choices to help explain sustaining cognitive status over a relatively short period of time, one year, we drew on a dataset that includes 4,620 community residing elders in independent housing sites in 24 US states. As residents of housing environments that belong to the COLLAGE consortium, all older adults were offered the opportunity to participate in the COLLAGE program and be assessed at baseline and one year later using two of interRAI's tools: the interRAI Community Health Assessment and interRAI Wellness tool [39]. Deidentified research copies of these data are maintained by interRAI and the study received approval from Institutional Review Board (IRB), Hebrew SeniorLife, Institute for Aging Research.

Assessors trained in the use of the interRAI instruments completed all assessments. The data collection occurs in the course of a conversation between the assessor and the elder and may include an informed family member as needed. The assessor training occurred separately at each site, but in each instance, the procedures followed models specified by interRAI [39]. Therefore, the reliability of the available data elements can be presumed to be quite good and consistent with data reported previously [9, 37, 38].

### 3.1. Cognitive Performance Scale

The dependent, outcome variable is cognitive decline of any magnitude over a one-year period, as measured by the Cognitive Performance Scale. This measure of cognitive ability consists of a subset of questions relative to decision making and communication [8]. The CPS scale has been shown to be a valid measure of cognitive status and has a strong relationship with the Mini Mental State Examination summary score [10]. The CPS has also been shown to be a key predictor of a person's current performance status as well as risk of change in the months to come [40, 41].

Because we focused on factors that might help to sustain cognitive ability over a limited, one-year time frame, we dichotomized the scale scores to decline or no decline for use as the dependent variable. We examined the association between sustaining one's cognitive status over a one-year period and the intellectual, physical, emotional, social, and spiritual variables within the assessment tools.

The goal was to identify factors associated with maintaining one's current cognitive status. Cognition in older adults is complex and affected by a multitude of factors. By examining assessment data from those who maintain their level of cognitive ability and those who decline in a one-year period, we seek to uncover potentially short-term, modifiable factors that may make a difference.

### 3.2. Domains of Wellness

Hettler's Domains of Wellness [12] served as the organizing framework for the independent variables.  Table 1 lists the independent variables used within each wellness domain. They reflect both wellness actions that the person can initiate (e.g., riding a bike or doing a crossword puzzle) and wellness passive states that the person experiences or is able to sustain in his/her daily life (e.g., sleeping through the night or not experiencing anxiety).

### 3.3. Data Analysis

All statistical analyses were completed using IBM SPSS Statistics 23 and all results with *p* < 0.05 were considered statistically significant. The analyses in each wellness domain proceeded through a multistep process. Baseline independent variables in each domain (scored in the most appropriate dichotomy format) were first assessed against the CPS-based cognitive decline dependent measure. Where significant, the univariate odds ratio (OR) is presented to gage the strength of the relationship.

Stepwise logistic regression was used for significant independent variables in each wellness domain to identify the resident characteristics within that domain that best identified older adults who are unlikely to experience cognitive decline. This step indicates whether each item in a wellness domain plays a protective role or whether there is an underlying single latent factor at work. Finally, we assessed three key baseline measures related to cognitive performance as possible controlling covariates to enter into the models: being not independent in decision making; short-term memory problem; and being not independent in being understood by others. The introduction of these covariates helps to ensure that the findings for each wellness domain were not a simple reflection of a person's baseline cognitive and communication status.

## 4. Results

The older adults in the independent housing sample (COLLAGE) came from 24 of the 50 US states and had their baseline assessment carried out between 2004 and 2013. Their average age was 81 years. In terms of basic demographics, 91% were white, 69% were female, 50% were married, and 54% lived alone. All elders resided in independent housing.

Functionally and cognitively, most sample members were independent at the time of the baseline assessment. From a functional perspective, 97% walked without the help of others and 90% could prepare a meal without assistance. The cognitive picture shows an equally independent population. Seventy-nine percent had a CPS of zero (intact cognition), 91% were independent in decision making, 84% had no problem with short-term memory, and 92% could fully understand others with whom they communicated. Over the one-year period between the baseline and follow-up assessment, 12% of the sample experienced a decline in cognition as measured by the CPS. For those who declined, the average CPS score declined by 1.9 points and of these persons 33% went down by one point, 49% by two points, and 18% by three or more points.

### 4.1. Physical Wellness Domain

Within this domain we first assessed problems with sleep, having difficulty falling asleep, obtaining too much sleep, and not having enough sleep, and, in each instance, these measures were not significantly related to a decline in cognitive performance.

We next examined baseline physical factors (Table 2) that might contribute to cognitive decline over the ensuing year. All seven of the individual activity and exercise physical wellness items were significantly related to cognitive performance change; those who engaged in these activities were less likely to experience cognitive loss (Table 2). Participation in biking, Pilates, yoga, and Tai Chi, swimming, hiking, or walking all had odds ratios less than one (thus they played a protective role with respect to subsequent cognitive decline). In addition, participating in 3 or more hours of exercise or physical activity in the last 3 days, participating in a formal exercise program in the last 3 days, and going outside at least 3 times in the last 3 days were all associated significantly with protection from cognitive decline. Multiple logistic regression results, with the addition of the baseline cognitive and communication covariates (both of which are themselves significantly related to cognitive decline, with odds ratios of 1.43 for memory and 1.78 for communication), suggest that four of the seven items (3 or more hours of physical activity, swimming, biking, and hiking/walking) continued to play a protective role after all factors in this domain plus the covariates were considered.

### 4.2. Intellectual Wellness Domain

The respondents reported their participation in five activities considered intellectually stimulating (Table 3). All demonstrated a significant univariate relationship and apparent protective influence on cognitive decline. These activities were computer interactions, crossword puzzles, arts and crafts, reading, and enrollment in educational courses. All showed odds ratios less than one (representing protective factors) with reading having the lowest ratio of 0.498. Multiple logistic regression results, with the addition of the baseline cognition and communication covariates, suggest that two of the five cognitively stimulating activities, computer activities, and reading played a protective role even after the other cognitive activities were considered.

### 4.3. Emotional Wellness Domain

Of the six individual emotional variables (Table 4), five had a significant protective effect on cognitive decline. The typical elders living in the community responded most positively in these areas and when they did, this sense of emotional wellness was a positive factor in sustaining cognitive independence. In the multivariate regression, with the addition of the baseline cognitive and communication covariates, two of the items remained significant, delighted/pleased with life and interest and pleasure in usual activities.

### 4.4. Social/Leisure Wellness Domain

Among the five social/leisure items, two appear to have a protective effect on cognitive decline in this study population, and both are significant in the univariate and multivariate models. A self-report of not feeling lonely (multivariate OR = .685, rates of 15.6 versus 11.3) and reports pursuing involvement in everyday life (multivariate OR = .576, rates of 15.3 versus 9.4) were protective of cognitive decline.

### 4.5. Spiritual Wellness Domain

Of the two measures in this wellness domain, both applied to about 95% of all persons. Ninety-six percent reported finding meaning in day-to-day life, while 95% said their spiritual needs are met. Only the latter played a protective role with respect to cognitive decline (multivariate OR = .467, rates of 18.4 versus 9.5).

## 5. Discussion

Over a one-year period, 12% of the elders in the sample of community residing elders experienced a loss in cognitive performance. Based on measures reflective of the Dimensions of Wellness model [12], a number of factors appeared to play a protective role for those sample members who did not decline. From our analyses, the physical and intellectual domains dominated in their association with cognitive decline. We positioned sleep within the physical domain. The reported results from previous studies regarding the relationship between sleep and cognitive decline showed some association [30–35]. In our analysis, there was no apparent relationship between sleep problems and a decline in cognitive performance thus showing some consistency with previous research.

Engaging in physical activity, formal exercise programs, and specific recreational activities such as biking, walking, and Pilates all had a protective effect on short-term cognitive decline. There are numerous studies between physical activity and current cognition performance affected by present and earlier lifestyle activities [17–20, 42]. A meta-analysis examining the relationship between physical activity and Alzheimer's disease reported multiple neurotrophic factors resulting from physical activity had a protective cognitive effect [43]. Our results were consistent with previous work and showed physical activity to have a strong protective effect on cognitive decline. James et al. [44] found elders whose lifespan was constricted to their homes were more likely to develop Alzheimer's disease. In our preliminary analysis, going outside the home 3 or more times in the past 3 days was protective of cognitive decline.

Our work also demonstrated the impact of intellectual wellness on cognitive decline. Active participation with computers, crossword puzzles, arts and crafts, and formal education courses all were protective factors. Others have found that participation in these activities in later years may compensate for limited formal education [20, 24, 25]. Not surprisingly, memory and communication problems were associated with increased risk for cognitive decline.

The remaining four dimensions of wellness demonstrated some limited associations with cognitive decline. Feeling valued, not feeling sad, feeling depressed, or feeling hopeless and expressing satisfaction with life as a whole were all protective of cognitive decline. Köhler et al. [28] found that older adults with depressive symptoms were more likely to develop cognitive impairment and an increased number of depressive symptoms increased the risk for cognitive decline. Wald [45] reported on the correlation between social isolation and dementia and there are several studies supporting the importance of personal and community relationships and social integration. In contrast, our results indicated only one element, not feeling lonely, was protective of cognitive decline. Finally, of the two measures of spirituality evaluated, feeling that one's spiritual needs were met played a protective role.

## 6. Limitations

There are many recognized causes for loss of cognitive function. These include structural changes in the brain, such as a brain tumor or a large subdural hematoma. More commonly, the gradual loss of high level functioning is attributed to Alzheimer's disease or another dementing illness. Yet even Alzheimer's disease is poorly understood; it remains uncertain whether the recognized changes in the brain associated with this condition are due to a single cause. Were there more than one cause, preventative measures might be more successful at some times rather than others.

The study sample was comprised of older adults residing in senior communities who were members of the COLLAGE consortium. Thus, all sample members were residing in some type of independent senior housing, be it a continuing care retirement community or below market housing. The sample did not include older adults who continue to live in privately owned homes or apartments.

We completed a secondary analysis of data gathered as a comprehensive set of measures on disease state, clinical complications, cognition, function, mood, social supports, environmental conditions, medication use, and health services use and extracted relevant factors. There was no opportunity to tailor questions specifically within each wellness domain. We did not examine data collection dates and therefore did not account for seasonal variations that could impact some influencing factors such as going out of home or participating in select, typically outdoor recreational activities such as biking or hiking.

## 7. Conclusions

Cognition and cognitive decline among older adults is a complex process. We presented our examination of cognitive decline within the context of a wellness model using Hettler's Dimensions of Wellness [12] and identified specific, daily activities that may provide protection from cognitive decline in the short-term. The Physical and Intellectual Dimensions of Wellness were prominent aspects in protection from cognitive decline but factors from the other dimensions, emotional, social/leisure, and spiritual, also offer some promise. While increase in physical activity previously has been shown to be beneficial, our results demonstrate specifically 3 or more hours of physical activity in 3 days, swimming, biking, and hiking/walking provided protection from cognitive decline. Similarly, intellectual stimulation is accepted as a reasonable preventive measure and our work singled out computer activities and reading as most beneficial. The other dimensions of wellness shed light on the emotional and social aspects of cognitive decline. Older adults in our sample were less likely to experience decline if they reported feeling delighted with their life and maintained interest and pleasure in their usual activities. Pursuing involvement in everyday life and not feeling lonely also were protective of decline. Finally, elders who reported their spiritual needs were met were less likely to experience cognitive decline.

Inherent in all of the dimensions are several mutable factors, amenable to targeted interventions to prevent or limit cognitive decline. These mutable factors offer a guide for older adults and their care providers in selecting targeted interventions to prevent decline.

The importance of attempting to decrease the rate of decline is highlighted by the expected rapid increase in the number of the elderly worldwide [46]. Even a modest decrease in the rate of decline of mental function would benefit even larger numbers of persons in the near future.

## Figures and Tables

**Table 1:** Wellness domain and associated assessment item.

Wellness domain	Assessment item
Physical, sleeping	Difficulty falling asleep Obtaining too much sleep Not obtaining enough sleep

Physical, exercise	Leaving house over the last 3 days
	Total hours of exercise or physical activity in last 3 days
	Exercise-related activity involvement
	Biking
	Pilates, yoga, Tai Chi
	Swimming/aqua fitness
	Hiking

Intellectual wellness/cognitive leisure	Computer activities
	Crossword puzzles
	Crafts or arts
	Educational courses
	Reading

Emotional wellness	Interest or pleasure in things you normally enjoy Not anxious, restless, or uneasy Not sad, depressed, or hopeless Stress does not have a negative effect on quality of life Feel valued Life satisfaction

Social/leisure wellness	Not lonely Have close friends in community Can count on friends for companionship Can count on friends for daily support Pursue involvement in life of community

Spiritual	Find meaning in day-to-day life Feel spiritual needs are being met

**Table 2:** Physical activity and CPS decline.

Item	% participation	% not participate with CPS2 decline	% participate with CPS2 decline	Univariate odds ratio (all sig at .05 or higher)	Multivariate odds ratio	Mult Sig	Mult 95% Con Int
Three or more hours of physical activity in past 3 days	45.0	14.0	9.5	.648	.670	.000	.553–.811
Out 3 times in past 3 days	75.9	14.8	11.1	.718			
Participation in exercise program in last 3 days	22.3	13.0	8.6	.635			
Biking	6.7	12.4	6.2	.464	.599	.037	.369–.970
Pilates, yoga, Tai Chi	10.4	12.4	8.4	.643			
Swimming	13.6	12.6	8.3	.625	.687	.016	.507–.931
Hiking or walking	39.2	13.9	8.9	.604	.717	.001	.585–.879

**Table 3:** Intellectual wellness.

Item	% participation	% participation with no CPS2 decline	% participation with CPS2 decline	Univariate odds ratio	Multivariate odds ratio	Mul Sig	Mul 96% Con Int
Computer activities	46.3	12.5	7.3	.550	.593	.000	.446–.788
Crossword puzzles	70.3	12.2	8.8	.699			
Crafts or arts	29.3	10.8	8.4	.751			
Education courses	35.3	11.3	7.9	.670			
Reading	85.4	16.3	8.8	.498	.607	.003	.438–.841

**Table 4:** Emotional wellness and life continuity.

Item	% participation	% participation with no CPS2 decline	% participation with CPS2 decline	Univariate odds ratio	Multivariate odds ratio	Mul Sig	Mul 95% Con Int
Interest or pleasure in usual activities	88.4	15.9	11.5	.682	.636	.009	.454–.891
Pleasure in usual activities	96.2	14.2	11.9	.NS			
Not anxious	91.4	16.2	11.6	.676			
Not sad	92.1	19.2	11.6	.630			
Delighted/pleased with life	64.7	12.3	9.0	.700	.771	.049	.596–.998
Feels valued	95.9	16.7	9.7	.537			
